# Knowledge and Attitudes About Breast Cancer in Limpopo, South Africa

**DOI:** 10.1200/JGO.2016.008102

**Published:** 2017-02-08

**Authors:** Lydia A. Trupe, Anne Rositch, Lindsay Dickerson, Su Lucas, Susan C. Harvey

**Affiliations:** **Lydia A. Trupe**, University of Cape Town School of Public Health and Family Medicine, Cape Town; **Su Lucas**, University of Witwatersrand School of Medicine and Chris Hani Baragwanath Academic Hospital, Johannesburg, South Africa; **Anne Rositch**, Johns Hopkins Bloomberg School of Public Health; **Lindsay Dickerson** and **Susan C. Harvey**, Johns Hopkins University School of Medicine; and **Susan C. Harvey**, Johns Hopkins Hospital, Baltimore, MD.

## Abstract

**Purpose:**

Breast cancer survival is unacceptably low in many low-resource settings, including rural South Africa, where access to screening and treatment services is limited. To describe the context for implementing an early detection program, we assessed knowledge and attitudes toward breast cancer risk, early detection, and treatment.

**Methods:**

We conducted a cross-sectional survey among 243 women presenting to Hlokomela Clinic in Hoedspruit, South Africa, during April and May 2016. We used quantitative and qualitative analyses to determine levels of knowledge of risk factors, symptoms, and treatment of breast cancer, as well as experience with and attitudes toward detection and treatment methods.

**Results:**

Thirty-one percent of women correctly identified at least six of 12 risk factors for breast cancer, and 53.1% identified breast lumps as an important symptom. Although > 97% of women stated that self–breast examination and early detection were highly important and that they would seek care for changes in their breasts, only 33.3% of women reported performing self–breast examination, and only 24.3% reported receiving a clinical breast examination. Age and education were not associated with knowledge, and level of knowledge did not predict care-seeking behaviors or attitudes.

**Conclusion:**

Although women demonstrated moderate levels of knowledge of breast cancer symptoms and risk factors and the importance of early detection, few women reported seeking services. These data demonstrate sufficient levels of knowledge and positive attitudes toward care seeking and suggest both a need and readiness for increased access to cost-effective services to facilitate early diagnosis and improved outcomes.

## INTRODUCTION

Breast cancer is the most commonly diagnosed cancer in women in both high- and low-resource settings.^1^ Although widely recognized as a significant public health concern in developed nations, breast cancer is also becoming an increasingly urgent issue in low- and middle-income countries (LMICs). Both incidence and mortality rates in LMICs have dramatically increased over the past few decades, and a majority of deaths resulting from breast cancer now occur in developing nations.^2^ In South Africa, breast cancer incidence has doubled in the past 20 years and now accounts for > 20% of all female cancers.^3,4^

Despite rapidly increasing incidence rates, early detection and treatment remain extremely limited in LMICs, and these countries face significantly higher mortality rates than high-income countries. The average 5-year survival in Africa is half of that in the United States (50% *v* 98.6%), and the age-standardized mortality rate for breast cancer in South Africa is 16.5 in 100,000, as compared with 14.1 in 100,000 in the United States.^3,5-7^

A primary cause of poor breast cancer survival in LMICs, including South Africa, is poor access to and uptake of early detection and cancer care. There is a paucity of research on breast cancer awareness and care-seeking behaviors in the rural provinces of Limpopo and Mpumalanga, South Africa. Therefore, we aimed to describe knowledge of and attitudes toward breast health in rural South Africa to determine the need and readiness for an early detection program in this population.

## METHODS

### Study Population and Design

We conducted a cross-sectional study at the Hlokomela Clinic in Hoedspruit, South Africa, from April to May 2016. The clinic serves both the Maruleng municipality in the Limpopo province and the Bushbuckridge municipality in the Mpumalanga province. The site was chosen because of its nonprofit status and long-standing positive presence in the community; the clinic population is demographically representative of our target population (ie, African women living in rural areas). Participants were sampled consecutively; each woman age ≥ 18 years was approached for inclusion.

Data collectors were members of the local community with experience in lay counseling and basic data collection, trained in both research methods and human research ethics and fluent in English, Xitsonga, and Northern Sotho (Sepedi). Date collectors collected written informed consent using a participant information sheet in the participant’s language of choice (English, Xitsonga, or Northern Sotho). Data collectors then verbally administered a five-page questionnaire in a quiet, private room. The five-part survey took approximately 10 minutes to administer and contained questions on demographic information, including age and level of education; personal and family histories of breast cancer; experience with breast cancer screening; knowledge of breast cancer risk and protective factors and symptoms; and knowledge of and attitudes toward breast cancer treatment. To assess knowledge of breast cancer risk and protective factors, participants were asked to state whether each of 12 factors increased the risk of breast cancer, decreased the risk of breast cancer, or had no impact (Table 1). We assigned a binary score of correct or incorrect to each response and totaled the percentage of risk factors or protective factors that participants correctly identified. Knowledge of and attitudes toward breast cancer treatment were assessed qualitatively, with participants asked to answer a series of open-ended questions. Ethical approval for this study was granted by the University of Witwatersrand Human Research Ethics Committee.

**Table 1:**
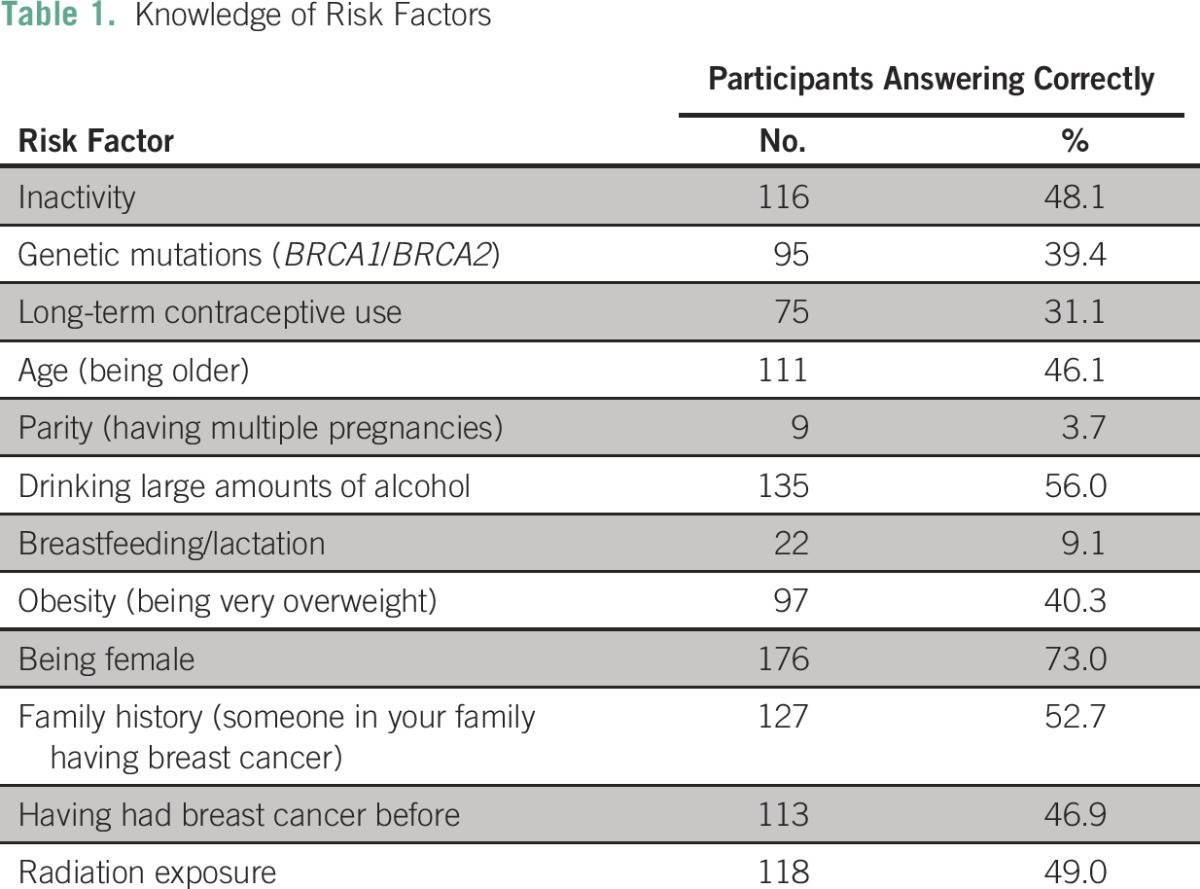
Knowledge of Risk Factors

### Data Analysis

Descriptive statistics are reported to describe the demographics of the study population. Percentages are reported for categorical variables including personal and family histories of breast cancer, screening and treatment histories, and knowledge of risk factors. We determined mean knowledge scores and categorized participants as having high or low knowledge on the basis of whether they were able to correctly identify ≥ 50% of the 12 risk or protective factors.

Knowledge of symptoms and treatment and attitudes toward care seeking were assessed using qualitative methods. Qualitative analysis was conducted by coding and categorizing responses to open-ended questions. There were a total of three open-ended questions: “list any symptoms of breast cancer that you know of”; “if you think there is treatment available for breast cancer in South Africa, please explain what you understand that treatment to be”; and “please state any reason you can think of why you might not contact a doctor if you noticed changes in your breasts.” We categorized similar responses and reported totals for each symptom, treatment method, and reason for deferral that we identified.

One-way analysis of variance was used to compare mean knowledge scores between demographic groups. χ^2^ tests were used to determine associations between level of knowledge and self-reported action. All data analyses were conducted using STATA software (version 13.1; STATA, College Station, TX), and tests of significance were two tailed at α = 0.05.

## RESULTS

We interviewed a total of 243 black African women between the ages of 18 and 68 years, with a mean age of 38 years (standard deviation, 9.7 years). A majority (59.0%) of respondents had an education level lower than grade 11, and 4% had a university or postgraduate degree (Table 2). The self-reported prevalence of breast cancer in the population was 1.2%; all of these women reported having received treatment. More than one third of women (33.8%) reported knowing either a family member or community member who had been diagnosed with breast cancer, including 7.0% who reported having a family member with breast cancer. Of women who reported knowing someone with breast cancer, 42.0% knew at least one person who had died as a result of breast cancer.

**Table 2:**
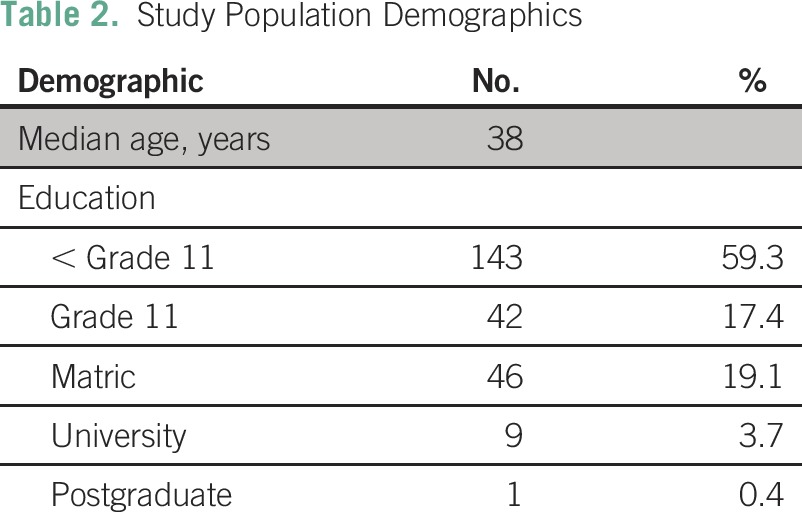
Study Population Demographics

### Knowledge of Breast Cancer Risk Factors, Symptoms, and Treatment

Respondents demonstrated moderate levels of knowledge with regard to breast cancer risk factors, symptoms, and treatment. Nearly one third of women (31.3%) were able to correctly classify six of 12 risk factors (Table 1). Thirty-two percent of women demonstrated low understanding of risk factors, correctly identifying fewer than four risk or protective factors. On average, women were able to correctly classify 41.3% (standard deviation, 22.8%) of risk factors. Just over half of women (53.1%) were aware that a lump in the breast was a symptom of breast cancer. The second most frequently cited symptom was pain in the breast (23.9%), and the third was inflammation of the breast, with 23.9% of women listing this symptom.

In comparison with symptoms and risk factors, respondents had lower knowledge of breast cancer treatment, with 38.7% stating that they were unaware of the methods of treatment, and 2.9% stating that breast cancer is not treatable. Less than one third (31.3%) of respondents stated that medicine or pills are used for treatment, although only 1.7% specified chemotherapy. Mastectomy (25.9%) and lumpectomy (9.1%) were the second and third most commonly identified treatment methods (Table 3).

**Table 3:**
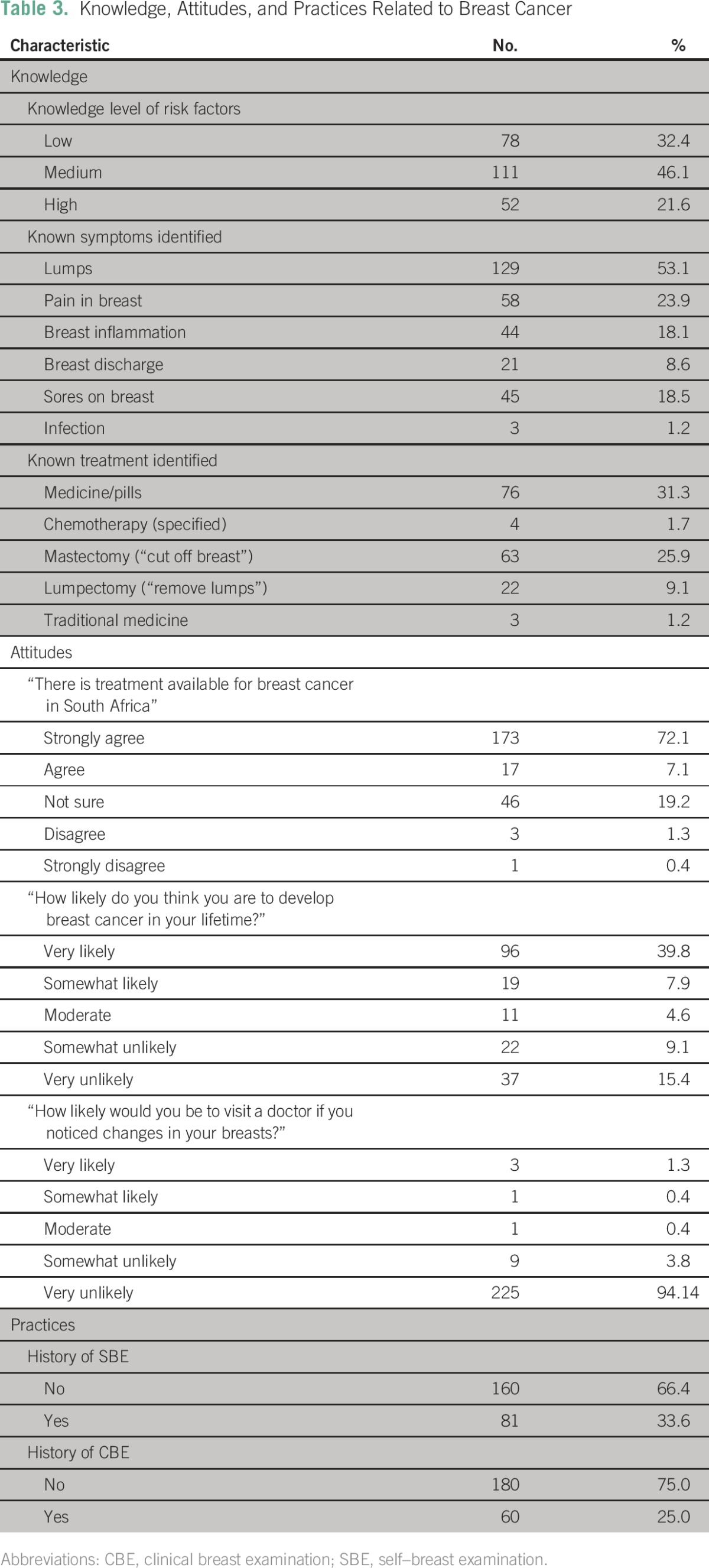
Knowledge, Attitudes, and Practices Related to Breast Cancer

### Attitudes Toward Care Seeking

Women in our population demonstrated strong positive attitudes toward care seeking, with > 97% of respondents stating that it was important to check their breasts regularly for breast cancer, that it was important to go to a physician or other care provider if they felt something abnormal in their breasts, and that they would be likely to go to a physician or other care provider should they notice changes in their breasts. A majority of women (67.1%) stated that they believed they would survive breast cancer if detection occurred early, whereas only 6.2% of women thought that they would be very likely to survive breast cancer if it was detected late. Women significantly overestimated their personal risk of breast cancer, with 47.7% responding that they were either very likely or somewhat likely to develop breast cancer in their lifetimes. The most commonly reported potential reason for not seeking care was fear of death or of having the breast cut off, although this was expressed by only 3.3% of respondents. Other potential reasons for not seeking care included lack of money, perceived poor quality of care at health facilities, long queues (particularly at public hospitals), and a belief that breast pain was not an important symptom. These reasons were only reported by a small minority of respondents (Table 3).

### Access to Care and Care-Seeking History

In contrast to high levels of care-seeking intention, only 24.8% of women reported having ever received a clinical breast examination (CBE), and only 33.3% of women reported having ever conducted a self–breast examination (SBE; Table 3). Increased levels of knowledge did not predict care-seeking behaviors. There were no correlations between risk factor knowledge and reported history of conducting SBE (*P* = .43) or age (*P* = .10). Those with greater than grade 11 education did have a higher mean score for risk or protective factor identification (43.5% *v* 39.7% for those with ≤ grade 11 education), but this did not meet statistical significance (*P* = .20).

## DISCUSSION

This study is the first to our knowledge to report data on knowledge, attitudes, and practices related to breast cancer in rural South Africa. Our findings generally echo those of similar studies conducted in urban and periurban settings in South Africa as well as other LMIC settings. Our results support findings from the Western Cape province that South African women perceive breast cancer to be both common and curable if detected early.^8^ Despite reported positive attitudes toward care seeking and higher levels of knowledge in comparison with those in other LMIC settings, our study identified an intention–action gap with respect to breast cancer care seeking in this population, indicating the need for increased access to early detection and treatment services.

Knowledge of breast cancer risk factors was significantly higher than knowledge levels reported in similar studies across Africa.^9^ Conversely, women in our study population demonstrated lower levels of knowledge of breast cancer symptoms than those surveyed in the Western Cape, as well as lower levels of SBE (33.3% *v* 65.0%) and CBE (24.8% *v* 62.0%).^8^ Although reported family and personal histories in our population were lower than those in previous South African studies, the relatively high proportion of women reporting knowledge of a family or community member with breast cancer (33.8%) suggests breast cancer prevalence rates similar to those seen in high-income countries.^3^

In previous reports on LMICs, delays in the decision to seek care have been reported to be the result of cultural influences on the decision to act, including fatalism, fear of stigma, and preference for traditional healers.^10^ Although women in both rural and urban or periurban settings in South Africa demonstrate an intention–action gap with regard to breast cancer care seeking, women in our study did not report the same justifications for delay in care seeking as their urban counterparts. Krombein and De Villiers^8^ found that the most commonly cited reasons for choosing not to seek care among women in the Western Cape included a fear of being diagnosed with breast cancer, insufficient knowledge, pain of the procedure, and cost. Although a small proportion of women in our population identified these same barriers, a vast majority of women did not report any reason for delay in seeking care. The comparatively high levels of knowledge and positive attitudes toward care seeking identified in our study suggest that structural barriers, including poor access to care, are responsible for limited breast care use in this population.

In low-resource and economically transitioning countries, breast cancer control can be conceptualized as a spectrum of stages or implementation phases from CBE to advanced imaging, which may include mammography and breast magnetic resonance imaging. Although SBE and CBE may be the only feasible options in low-resource settings, when combined with education about the early signs and symptoms of breast cancer, these may be important tools to facilitate early detection.^11^ However, it is clear that, for a screening program to improve outcomes, an accessible treatment program must also be in place. There are several ways this could be achieved, again ranging from promising nonsurgical options such as ablative techniques to traditional treatments with surgery, chemotherapy, and/or radiation therapy.^12^ A similar paradigm, where alternative screening and treatment options are acceptable in a given context on the basis of available resources and capacities, has been put forth by the WHO for cervical cancer prevention.^13^ Thus, research is needed to provide an evidence base for alternative strategies to detect and treat breast cancer that may be better suited to low-resource settings. The present research will be used to inform further work by the authors on alternative screening options, including breast ultrasound, as well as nonsurgical treatment options for low-resource settings.

There are limitations to this study that must be considered when interpreting the results. First, the sample size was relatively small and may not be representative of all women in the rural setting, because women were enrolled while seeking care at a primary health care clinic. Our study site, Hlokomela Clinic, also uses a peer health education model, in which trained community members (called nompilos) disseminate health information throughout the community. Thus, the women in this population may be both more educated about breast health and more comfortable with seeking health care than the general population in rural South Africa. The relative homogeneity of our population (with > 95% of participants having ≤ high school education) meant that we were unable to identify variations by education level. Future research should aim to determine whether differences in primary versus high school education predict levels of knowledge in low-resource settings, as well as what roles socioeconomic status and access to health services play in determining knowledge, attitudes, and practices. As is the case in all cross-sectional surveys, our study is subject to both misclassification and social desirability bias. Data on family and personal histories as well as history of early detection practices were dependent on self-report and thus may have been under- or over-reported, which is the standard in this region and generally in LMICs where no formal medical data collection exists. Social desirability bias may have led to over-reporting of care-seeking intentions. The adaptation of our survey from English for a low-literacy, Tsonga- and Sepedi-speaking population may have also led to misclassification. Women were asked in the survey how likely they were to visit a physician. After completion of the survey, it became clear that women in the study population typically distinguish between physicians and other care providers (eg, the general care practitioners and nurses who practice at local clinics). We were unable to assess familiarity with and history of screening ultrasound or mammogram, because of a translation error in the survey. Future research should seek to identify current practices with regard to these technologies in South Africa.

Women in our study population demonstrated moderate levels of knowledge about breast cancer risk factors and symptoms, recognized the importance of early detection for breast cancer treatment, and demonstrated positive attitudes toward care seeking for breast cancer despite low access to care in the region. Our data suggest that implementation of an early detection and treatment program has a high likelihood of acceptability. Further research is needed on the feasibility and impact of such a program.
